# Long-term mental health outcomes among healthcare workers following COVID-19 infection and frontline work: evidence of additive and non-additive patterns in Kazakhstan

**DOI:** 10.1186/s12889-026-28195-2

**Published:** 2026-06-25

**Authors:** Dinara Ospanova, Kundyzay Tobzhanova, Lyazzat Alibekova, Ainur Seitmanova, Galiya Bilibayeva, Zhanar Ibraimzhanova, Raushan Kurmankhanova, Ulbossyn Shokay, Roza Alekesheva, Meruyert Suleimenova

**Affiliations:** 1 Department of Nursing, Al-Farabi Kazakh National University, Almaty, Kazakhstan; 2 Department of Health Policy and Organization, Al-Farabi Kazakh National University, Almaty, Kazakhstan; 3 High Medical College of the Almaty Public Health Department, Almaty, Kazakhstan

**Keywords:** Burnout, Healthcare workers, COVID-19, Anxiety, Mediation, Central Asia

## Abstract

**Background:**

Long-term mental health outcomes of COVID-19 related exposures among healthcare workers remain poorly characterized, particularly in Central Asia.

**Methods:**

We conducted a cross-sectional comparative study between June and August 2025 among 2715 healthcare workers. Participants completed validated measures of BAT-33, GAD-7, PHQ-9, and EQ-5D-5 L with EQ-VAS. Main and interaction effects were examined by two-way ANСOVA and associations were decomposed into direct and indirect components using path analysis with bootstrap confidence intervals.

**Results:**

COVID-19 infection was associated with higher burnout, while the COVID × Red Zone interaction was not significant, indicating an additive pattern. In contrast, significant interactions were observed for anxiety, depression, and quality of life, suggesting higher psychological symptoms among workers with dual exposure. Red Zone work showed a statistically significant but small association with burnout. In mediation analyses, anxiety and depression statistically accounted for 39.7% of the variance in burnout (R^2^), suggesting these affective states are strongly associated associated with both pandemic-related exposures and burnout.

**Conclusions:**

Pandemic-related occupational and personal exposures remained statistically associated with mental health outcomes among healthcare workers 3–5 years after COVID-19, with burnout showing an additive pattern and anxiety and depression showing non-additive deviations. Within our sample, the dual-exposed subgroup showed approximately 2-fold higher prevalence of clinically significant symptoms of anxiety and depression than unexposed colleagues. These findings identify a subgroup that may warrant continued monitoring in future longitudinal research with objective exposure verification, before specific clinical or policy recommendations can be advanced. This is among the first large-scale studies examining post-pandemic psychological outcomes among healthcare workers in Central Asia.

**Supplementary Information:**

The online version contains supplementary material available at https://doi.org/10.1186/s12889-026-28195-2.

## Background

The COVID-19 pandemic strained health systems worldwide, with lasting psychological consequences for healthcare workers. Early estimates from the World Health Organization suggest that up to 180,000 health and care workers may have died from COVID − 19 in the first year of the pandemic, and global surveys consistently document high levels of stress, burnout, anxiety, and depression across the healthcare workforce [1]. The question is not only whether psychological symptoms emerged during the pandemic, but why they persist years later. One likely contributor is post-acute COVID-19 sequelae, fatigue, cognitive difficulties, and mood changes that many patients continue to experience well after acute infection [2]. Beyond biological effects, the occupational context matters: repeated exposure to patient deaths, moral distress from impossible resource decisions, and sustained uncertainty about personal safety can exhaust even robust coping systems. From a Conservation of Resources perspective [3, 4], the problem is not simply that stressors were intense, but that the resources depleted during the pandemic which are time to recover, collegial support, sense of professional competence, were never fully restored. When staffing shortages and workload pressures continue post-pandemic, there is no recovery window. Taken together, these factors suggest that psychological consequences of the pandemic are better understood as ongoing conditions than as delayed reactions to a past event. Meta-analytic evidence indicates that burnout, anxiety and depression affected more than one-third of healthcare workers during the pandemic, raising concerns about workforce sustainability and patient safety [5].

Burnout is closely linked to affective symptoms. Higher burnout levels correlate strongly with heightened anxiety and depression, and healthcare workers experiencing burnout tend to score substantially higher on the GAD-7 and PHQ-9 compared with non-burnout peers [6]. These patterns highlight the need for organizational responses that reduce workload, strengthen institutional support, and improve access to mental-health resources [7]. Effective responses require action at individual, organizational, and system levels [8].

Several theoretical perspectives help explain how pandemic-related stressors may accumulate. The Job Demands–Resources (JD-R) model proposes that burnout develops when job demands chronically exceed available resources [9, 10]. If Red Zone work and personal COVID-19 infection contribute to a shared underlying pathway (for example, cumulative resource depletion), a non-additive pattern may be observed on the risk difference scale [11]. Conversely, if these exposures operate through largely independent pathways, their combined effect would approximate the sum of their separate effects. Within a COR framework, dual exposure may produce non-additive effects because each stressor further depletes a resource base already compromised by the other, amplifying strain beyond what either exposure alone would generate [3, 4]. Given the novelty of examining this specific combination, interaction effects are treated as an exploratory question rather than a confirmatory test. Evidence from COVID-19 research suggests that pandemic-related stressors may accumulate either additively or interactively. For example, some studies have found linear increases in emotional exhaustion with a greater number of COVID-related stressors [12], consistent with an additive pattern.

At the same time, stress-sensitisation models suggest that prior or concurrent stress exposure can lower the threshold for adverse affective responses to subsequent stressors, so that simultaneous exposures may produce a disproportionate, non-additive increase in psychological symptoms [13, 14]. Respectively synergistic amplification has been reported for occupational stressors during the pandemic [15]. In this context, understanding whether Red Zone work and personal COVID-19 infection exert additive or non-additive effects is important for identifying vulnerable subgroups and planning prevention strategies.

Red Zone work fits naturally within the JD-R framework as an elevated job demand. Personal COVID-19 infection is less straightforward to situate in this model, and we draw on Conservation of Resources theory [3] to fill this gap. COR theory holds that psychological strain arises not just from excessive demands, but from the loss or threatened loss of valued resources. Contracting COVID-19 represents a multi-layered resource loss: physical health and energy are compromised during illness; social connection is disrupted by quarantine; professional confidence may falter if work capacity is reduced; and uncertainty about long-term health may sustain a state of chronic resource vigilance. Crucially, COR theory predicts that resource losses compound rather than simply accumulate, which means that early losses reduce the individual’s capacity to protect remaining resources, creating what Hobfoll termed a loss spiral [4]. This suggests that workers exposed to both Red Zone work and personal COVID-19 infection may face compounded strain exceeding what either exposure alone would be expected to produce.

During the pandemic, healthcare workers were exposed to multiple, often overlapping stressors. Occupational exposures include direct patient care in COVID-19 units (often designated as “Red Zones” requiring enhanced personal protective equipment), heightened infection risk, increased workloads, staff shortages, and moral distress from witnessing patient suffering and death [16, 17]. Personal exposures encompass the experience of contracting COVID-19 themselves, with attendant physical symptoms, recovery challenges, and psychological aftermath.

Despite extensive research documenting elevated mental health problems among healthcare workers during COVID-19, less is known about how different types of exposures -occupational versus personal-independently and jointly affect psychological outcomes. Most studies have examined these exposures in isolation or have simply compared frontline versus non-frontline workers [12]. However, recent evidence suggests that both COVID-19 related occupational stressors and personal infection history are independently associated with adverse mental health outcomes such as insomnia, with potential additive or non-additive effects [18]. The question of whether dual exposure-experiencing both personal COVID-19 infection and occupational Red Zone work-produces cumulative stress effects that exceed those of single exposures has not been systematically tested.

This is especially relevant in Central Asian countries like Kazakhstan, where healthcare system resources were strained during the pandemic, yet systematic research on healthcare worker mental health remains limited. Kazakhstan experienced a significant COVID-19 burden, leading to intensive mobilization of the healthcare workforce and the rapid conversion of facilities. The scale of the crisis in the country placed enormous pressure on local health services, requiring external organizational support and adaptation, particularly in managing co-morbidities like tuberculosis [19]. A recent multi-country study of healthcare workers in Kazakhstan, Kyrgyzstan, and Uzbekistan found that Kazakhstani healthcare workers reported depression (13.8%), anxiety (19.2%), and stress (5.1%) during the pandemic [20], which points to considerable mental health burden in this workforce. However, the specific contributions of different exposure types-and their potential interaction-have not been systematically examined in this population. Beyond examining direct effects of pandemic exposures on burnout, we also explored whether anxiety and depression are statistically associated with both pandemic-related exposures and burnout. The JD-R health impairment process [21] suggests that sustained demands produce psychological strain that often co-occurs with anxiety and depressive symptoms as well as with burnout, though the temporal ordering of these constructs cannot be established without longitudinal data. This view is consistent with research showing strong associations between affective symptoms and burnout in healthcare workers [22]. We recognize that these constructs are interrelated and that bidirectional influences are plausible, so we treat this part of the analysis as exploratory. Given the cross-sectional design, the mediation analysis is intended to describe patterns of association rather than to demonstrate causal sequences. Clarifying how occupational and personal COVID-19 exposures influence mental health is important for identifying vulnerable subgroups and tailoring preventive support. The aim of this study was to examine whether personal COVID-19 infection and Red Zone work independently or jointly influence long-term psychological outcomes among healthcare workers, and to determine whether their combined effects are additive or demonstrate statistical interaction.

## Methods

### Study design and participants

This was a cross-sectional comparative study based on exposure status. Participants were classified into four groups according to two naturally occurring binary exposure variables (COVID-19 infection: yes/no, Red Zone work: yes/no), forming a 2 × 2 exposure structure. No experimental manipulation or random allocation was performed. A factorial analytical framework was applied to examine the independent (main) and combined (interaction) effects of these exposure variables on mental health outcomes. Such an approach is appropriate in observational research when categorical predictors define mutually exclusive groups. The four groups (Control, Red Zone Only, COVID Only, Both) emerged from self-reported exposure histories. The “Control” group comprised participants who reported neither COVID-19 infection nor Red Zone work. Data were collected between June and August 2025 via an online survey distributed through hospital administrators and nursing supervisors across Kazakhstan.

### Recruitment and sampling

The survey link was distributed through multiple channels to maximize geographic coverage. Invitations were sent through hospital administrators and nursing supervisors of healthcare institutions across Kazakhstan. The link was also circulated through professional messaging groups (e.g., WhatsApp and Telegram) and professional social media networks used by healthcare workers. Because the survey was distributed through open professional networks, the exact number of individuals who received the invitation could not be determined; therefore, a precise response rate could not be calculated. Participants represented healthcare workers from multiple regions of Kazakhstan and from different types of healthcare facilities, including hospitals and outpatient clinics. As participation was voluntary, the sample should be considered a convenience sample and may be subject to self-selection bias. To reduce potential bias, the survey invitation was distributed broadly through multiple institutional channels rather than targeting specific departments or exposure groups. Participation rates varied across regions, which likely reflects differences in institutional engagement and local dissemination of the survey link. According to the national statistical yearbook of the Ministry of Health, Kazakhstan employed 81,285 physicians and 190,644 nursing staff across all healthcare agencies in 2023, giving a total eligible workforce of approximately 272,000 [23]. A total of 3,162 healthcare workers accessed the survey, representing approximately 1.2% of this workforce. All healthcare workers currently employed in clinical roles were eligible. The sample therefore represents a voluntary convenience sample rather than a stratified or probability sample.

Of 3,162 healthcare workers who accessed the survey (91.9% nurses, 8.1% physicians), 447 (14.1%) were excluded for incomplete exposure data, yielding a final sample of 2,715 (mean age 40.3 ± 12.6 years; mean work experience 15.6 ± 12.7 years). This distribution reflects the actual composition of the healthcare workforce in Kazakhstan, where nurses constitute the majority of clinical staff. Accordingly, findings should be interpreted primarily in the context of nursing professionals, and generalizability to physicians should be approached with caution. Sample size exceeded requirements for two-factor ANOVA with small effect (η²=0.02, α = 0.05, power=0.80), providing > 99% power [24].

Inclusion criteria: (1) currently employed in healthcare, (2) aged ≥ 18 years, (3) worked during COVID-19 pandemic (2020–2022), (4) able to provide informed consent. Exclusion criteria were incomplete responses on exposure variables or logically inconsistent responses. Participants were classified into four groups: (1) Control (no Red Zone work, no COVID-19 infection; *n* = 1,237); (2) Red Zone Only (*n* = 515); (3) COVID-19 Only (*n* = 428); (4) Both Exposures (*n* = 535). Survey completion time was approximately 15–20 min.

### Questionnaire and measures

The measures used in this study are presented below, grouped by their analytical role: the primary outcome (burnout), the mediators (anxiety and depression), a secondary outcome (health-related quality of life), and covariates. All instruments were administered in validated Russian-language versions commonly used in Kazakhstani healthcare settings and have been widely applied in prior psychological and occupational health studies in the region [25–30].

### Burnout Assessment Tool (BAT-33)

Occupational burnout was assessed using the 33-item BAT [31, 32], available freely for research (www.burnoutassessmenttool.be). The BAT-33 measures four dimensions (exhaustion, mental distance, emotional impairment, cognitive impairment) on 5-point scales (1 = never to 5 = always). For analysis, a mean total burnout score was calculated by averaging all 33 items (range 1–5), with higher values indicating greater burnout severity. Internal consistency was excellent (Cronbach’s α = 0.97).

### Generalized Anxiety Disorder scale (GAD-7) and Patient Health Questionnaire (PHQ-9)

Anxiety and depression were measured using validated versions of the GAD-7 [33] and PHQ-9 [34]. The GAD-7 comprises 7 items (range 0–21; cut-off ≥ 10 for clinical anxiety). The PHQ-9 comprises 9 items reflecting DSM-IV criteria (range 0–27; cut-off ≥ 10 for clinical depression), with higher scores indicating greater depression severity. Internal consistency: GAD-7 α = 0.95, PHQ-9 α = 0.95.

### Health-related quality of life (EQ-5D-5 L)

Health-related quality of life was assessed as a secondary outcome using the EQ-5D-5 L. The EQ-5D-5 L was used under a non-commercial license granted by the EuroQol Research Foundation (User Agreement ID: 51AA2445-3DEF-4A13-8AE4-4EB0A7CAFE7B) [35]. The instrument comprises five levels (mobility, self-care, usual activities, pain/discomfort, anxiety/depression), generating a Level Sum Score (LSS) and a Visual Analogue Scale (EQ-VAS; 0–100). Previous studies have demonstrated adequate psychometric performance of the EQ-5D-5 L in healthcare professionals and in general population samples [36–40]. Because no validated EQ-5D-5 L value set currently exists for Kazakhstan, we calculated a LSS by summing the five dimension levels (range 5–25; lower scores indicating better health status). The LSS approach preserves ordinal severity information without applying preference weights derived from external populations. Both EQ-LSS and EQ-VAS were analyzed as complementary indicators of health-related quality of life.

To evaluate applicability within our sample, we examined response distributions across dimensions and calculated internal consistency of the five dimensions. Cronbach’s α was 0.84 (95% CI: 0.83–0.85), and the average inter-item correlation was 0.51. Response distributions demonstrated variability across severity levels without evidence of extreme clustering. Detailed results are provided in Supplementary Table S3.

### Statistical analysis

The analytical strategy followed a structured sequence aligned with the study aims. First, two-way ANCOVA was used to examine the independent and combined effects of COVID-19 infection and Red Zone work on continuous outcomes (burnout, anxiety, depression, and quality of life), including tests of interaction effects. Second, post-hoc pairwise comparisons (Tukey HSD) were conducted to clarify differences between exposure groups. Third, logistic regression models were used to estimate the odds of clinically significant anxiety and depression (GAD-7 ≥ 10, PHQ-9 ≥ 10), providing a clinically interpretable complement to the continuous analyses. Finally, path analysis was used to examine whether anxiety and depression statistically accounted for the association between pandemic-related exposures and burnout. All models were adjusted for age, sex, work experience, education level, and profession.

Descriptive statistics were presented as mean ± SD for continuous variables and n (%) for categorical variables. Group differences were examined using one-way ANOVA (continuous outcomes) and χ² tests (categorical variables). Normality was assessed via histograms and Q–Q plots; given the large sample size, parametric tests were considered robust to moderate deviations from normality. Post-hoc pairwise comparisons for one-way ANOVA were performed using Tukey’s HSD correction.

To examine whether the combined effects of COVID-19 infection and Red Zone work deviated from an additive pattern, we conducted two-way analyses of covariance (ANCOVA) models with interaction terms. The interaction term tests whether the joint effect of both exposures differs from the sum of their independent effects. A statistically significant interaction indicates deviation from additivity, whereas a non-significant interaction is consistent with an additive pattern. To facilitate interpretation of interaction effects, we calculated the expected additive mean for the dual-exposure group as:

$$\begin{aligned}\mathrm{M}_\mathrm{additive}=&\mathrm{M}_\mathrm{control}+\left(\mathrm{M}_\mathrm{COVID}-\mathrm{M}_\mathrm{control}\right)\\&+\left(\mathrm{M}_\mathrm{RZ}-\mathrm{M}_\mathrm{control}\right).\end{aligned}$$

This calculation serves a descriptive and illustrative purpose only and does not constitute a separate statistical test; the formal test of non-additivity is provided by the interaction term in the ANCOVA model.

The deviation from additivity was defined as the difference between the observed dual-exposure mean and the additive prediction. Because the outcome variables were continuous symptom scores rather than binary risk indicators, epidemiological measures of additive interaction (e.g., Synergy Index or Attributable Proportion) are not typically applied to continuous symptom scales. Models were adjusted for age (mean-centered), sex, work experience (mean-centered), education level, profession (doctor vs. nurse), and region of residence. These variables were included as covariates to control for potential confounding effects. Type III sums of squares with orthogonal contrasts were used to estimate main and interaction effects. A significant COVID-19 × Red Zone interaction term was interpreted as evidence of non-additive effects. Effect sizes were expressed as partial eta-squared (ηp²), with thresholds of 0.01 (small), 0.06 (medium), and 0.14 (large) according to conventional guidelines [41]. Adjusted ANCOVA models were estimated using complete-case data. Three analytic samples were used depending on data availability. Post-hoc pairwise comparisons (Table 4) used the full sample (*N* = 2,715). Adjusted ANCOVA models (Table 3) excluded 71 participants (2.6%) with missing covariate values, resulting in *N* = 2,644, adjusted for age, sex, work experience, education level, profession, and region of residence - the same covariate set as the Logistic regression models (Table 5).

Binary logistic regression models were used to identify predictors of clinically significant anxiety (GAD-7 ≥ 10) and depression (PHQ-9 ≥ 10). Model coefficients were estimated on the log-odds scale and exponentiated to yield odds ratios (OR = exp(β)), which are reported with 95% CI. Binary outcomes were defined using established clinical cut-offs (GAD-7 ≥ 10; PHQ-9 ≥ 10) validated for identifying clinically significant anxiety and depression symptoms [33, 34]. This categorical approach complements analyses of continuous scores by enabling estimation of the odds of clinically relevant anxiety and depression. Burnout (BAT-33) and health-related quality of life (EQ-5D-5 L) were not subjected to logistic regression because no universally accepted clinical cut-off exists for the BAT-33 total score in this population, and the EQ-5D-5 L LSS is an ordinal composite measure for which binary categorisation would result in loss of information without a clinically validated threshold.

Mediation analysis was conducted using path analysis in lavaan (v0.6-20). Indirect effects were computed as the product of constituent path coefficients and evaluated using bias-corrected bootstrap confidence intervals (2,000 resamples). We report unstandardized indirect effects with percentile bootstrap 95% CIs, together with standardized estimates (std.all) to facilitate interpretation. Path analysis coefficients in the results section refer to standardized values unless specified otherwise. Model estimation used maximum likelihood with robust standard errors where appropriate.

Anxiety and depression were modeled as parallel mediators because both represent partially overlapping but distinct affective constructs. Given the cross-sectional design, no temporal precedence between anxiety and depression could be established. Specifying a serial mediation model would therefore imply causal ordering not supported by the data.

We note that cross-sectional mediation cannot establish temporal ordering or test causal pathways [42]. The analysis therefore serves to decompose observed associations into direct and indirect components, providing descriptive information that may guide future longitudinal research.

All analyses were conducted in R (version 4.4) using the dplyr, rstatix, lavaan, and gt packages. Two-tailed *p* < .05 was considered statistically significant.

## Results

Table 1 summarizes the main characteristics of the 2,715 participants. The sample consisted of 2,491 nurses (91.7%) and 224 physicians (8.3%), with physicians more frequently represented in the COVID-infected groups (e.g., 16.4% in COVID Only vs. 4.4% in Control; *p* < .001). The Control group was the youngest (36.4 ± 12.9 years), while the RZ Only, COVID Only, and Both groups were older (42.8 ± 11.1; 43.8 ± 12.3; and 44.2 ± 10.8 years, respectively). Women comprised the majority across all categories (91.0–95.0%). Work experience followed the same pattern: 11.7 ± 12.2 years in the Control group versus 18.0–19.7 years in the exposure groups. Married participants were less common in the Control group (53.2%) compared with the exposure groups (59.8–61.2%). Higher education was also more frequent among exposed workers, rising from 16.9% in the Control group to 34.0% among those with both exposures. Most respondents were from the South (88.0–96.2%), but the proportion from the North/East and West was higher in the Both group (7.9% and 4.1%).

**Table 1:** Demographic characteristics of the study sample by exposure group

Group	Control	RZ Only	COVID Only	Both	Total	*p* - value
n	1237	515	428	535	2715	
Age (Mean ± SD)	36.4 ± 12.9	42.8 ± 11.1	43.8 ± 12.3	44.2 ± 10.8	40.3 ± 12.6	< 0.001
Years in healthcare (Mean ± SD)	11.7 ± 12.2	18.0 ± 11.9	18.7 ± 13.3	19.7 ± 11.7	15.6 ± 12.7	< 0.001
Female, n (%)	1175 (95.0%)	486 (94.4%)	401 (93.7%)	486 (91.0%)	2548 (93.9%)	0.015
Married, n (%)	658 (53.2%)	312 (60.6%)	262 (61.2%)	320 (59.8%)	1552 (57.2%)	0.002
Higher education (Bachelor+, n (%))	209 (16.9%)	104 (20.2%)	131 (30.6%)	182 (34.0%)	626 (23.1%)	< 0.001
Region						
South, n (%)	1190 (96.2%)	480 (93.2%)	397 (92.8%)	471 (88.0%)	2538 (93.5%)	< 0.001
North/East, n (%)	28 (2.3%)	20 (3.9%)	30 (7.0%)	42 (7.9%)	120 (4.4%)	< 0.001
West, n (%)	19 (1.5%)	15 (2.9%)	1 (0.2%)	22 (4.1%)	57 (2.1%)	0.008
Profession						< 0.001
Nurse, n (%)	1183 (95.6%)	493 (95.7%)	358 (83.6%)	457 (85.4%)	2491 (91.7%)	
Doctor, n (%)	54 (4.4%)	22 (4.3%)	70 (16.4%)	78 (14.6%)	224 (8.3%)	

Table 2 shows clear differences in mental health indicators across the four exposure groups. Burnout (BAT-33) was lowest in the Control group (1.67 ± 0.68) and gradually higher in the COVID Only (1.85 ± 0.74) and Both groups (1.96 ± 0.91), with a smaller change in the RZ Only group (1.72 ± 0.69; F = 21.29, *p* < .001). Anxiety and depression showed a comparable gradient. Participants with both exposures reported the highest levels (GAD-7: 5.08 ± 5.87; PHQ-9: 6.05 ± 6.76), approximately 1.5–2 times higher than in the Control group. Health-related quality of life showed a parallel trend. On the EQ-5D-5 L LSS, the both group reported more problems (7.95 ± 4.19) compared with the Control group (6.70 ± 3.01; F = 26.58, *p* < .001). Internal consistency of the EQ-5D-5 L dimensions was good (Cronbach’s α = 0.84; 95% CI: 0.83–0.85) (Table S4). Response distributions demonstrated variability across severity levels, without extreme clustering at higher severity categories. EQ-VAS scores were lowest in the dual-exposure group (71.50 ± 22.85) compared with the control group (76.14 ± 23.18), with significant differences across groups (F = 11.00, *p* < .001).

**Table 2:** Burnout, Depression, Anxiety, and Quality of Life by Exposure Status (*N* = 2,715)

Outcome	Control (*n* = 1,237)M ± SD	RZ Only (*n* = 515)M ± SD	COVID Only (*n* = 428)M ± SD	Both (*n* = 535)M ± SD	F	*p*	ηp²
BAT-33 Burnout	1.67 ± 0.68	1.72 ± 0.69	1.85 ± 0.74	1.96 ± 0.91	21.29	< 0.001	0.023
GAD-7 Anxiety	2.91 ± 4.30	2.86 ± 4.13	4.14 ± 4.97	5.08 ± 5.87	32.01	< 0.001	0.034
PHQ-9 Depression	3.72 ± 5.33	3.37 ± 4.93	4.98 ± 5.57	6.05 ± 6.76	28.60	< 0.001	0.031
EQ-5D-5 L LSS	6.70 ± 3.01	6.39 ± 2.39	7.15 ± 2.80	7.95 ± 4.19	26.58	< 0.001	0.035
EQ-VAS QoL	76.14 ± 23.18	78.98 ± 23.09	73.19 ± 22.60	71.50 ± 22.85	11.00	< 0.001	0.014

Post-hoc tests showed the Both groups differed most consistently from the other groups. Its burnout scores were significantly higher than those of the Control and RZ Only groups (both *p* < .001) and moderately higher than the COVID Only group (*p* = .019). The RZ Only and Control groups did not differ (*p* = .246). Full pairwise comparisons, including CI and effect sizes, are available in Supplementary Table S1.

Table 3 presents the adjusted main effects of COVID-19 infection and Red Zone work after controlling for age, sex, work experience, education level, profession, and region. Personal COVID-19 infection showed consistent associations across outcomes, with significant main effects for burnout, anxiety, depression, EQ-5D-5 L LSS, and EQ-VAS (all *p* < .001). Effect sizes were small but consistent. The independent effect of Red Zone work was limited. After adjustment, it remained statistically significant for burnout (*p* = .016) and EQ-5D-5 L LSS (*p* = .045), but effect sizes were small (ηp² ≈ 0.002). Red Zone work was not significantly associated with depression or EQ-VAS.

Three of five outcomes showed significant COVID × RZ interactions after adjustment (LSS: F = 16.74, *p* < .001, ηp² = 0.006; PHQ-9: F = 6.71, *p* = .010, ηp² = 0.003; GAD-7: F = 4.32, *p* = .038, ηp² = 0.002), indicating that dual exposure was associated with worse outcomes than expected under an additive pattern for several measures. The interaction for EQ-VAS was not statistically significant after adjustment (F = 3.02, *p* = .082). Burnout remained the sole outcome without a significant interaction (F = 0.39, *p* = .532), consistent with an additive pattern for that domain.

**Table 3:** Two-way ANCOVA: Adjusted Main and Interaction Effects of Red Zone Work and COVID-19 Infection (*N* = 2,644) (Adjusted for age (mean-centered), sex, work experience (mean-centered), education level, profession, and region)

Outcome	Main Effect: COVID-19 Infection	Main Effect: Red Zone Work	Interaction: COVID × Red Zone
BAT-33 Burnout	F = 29.26, *p* < .001, ηp² = 0.011	F = 5.77, *p* = .016, ηp² = 0.002	F = 0.39, *p* = .532, ηp² < 0.001
GAD-7 Anxiety	F = 55.79, *p* < .001, ηp² = 0.021	F = 6.00, *p* = .014, ηp² = 0.002	F = 4.32, *p* = .038, ηp² = 0.002
PHQ-9 Depression	F = 51.25, *p* < .001, ηp² = 0.019	F = 2.90, *p* = .088, ηp² = 0.001	F = 6.71, *p* = .010, ηp² = 0.003
EQ-5D-5 L LSS	F = 43.79, *p* < .001, ηp² = 0.016	F = 4.01, *p* = .045, ηp² = 0.002	F = 16.74, *p* < .001, ηp² = 0.006
EQ-VAS Quality of Life	F = 34.32, *p* < .001, ηp² = 0.013	F = 0.21, *p* = .649, ηp² < 0.001	F = 3.02, *p* = .082, ηp² = 0.001

Table 4 presents pairwise comparisons across all outcomes. A consistent pattern emerged: the RZ Only group did not differ significantly from the Control group on any outcome (all *p* ≥ .086, all |d| ≤ 0.12), whereas the Both and COVID Only groups showed significantly elevated symptom levels compared with Control on burnout, anxiety, and depression (all *p* < .001). The Both group differed significantly from the COVID Only group on anxiety (*p* = .012), depression (*p* = .018), and EQ-5D-5 L LSS (*p* < .001), indicating that dual exposure was associated with worse outcomes than COVID-19 infection alone.

**Table 4:** Post-hoc pairwise comparisons (Tukey HSD) across all outcomes

Comparison	BAT-33 (Burnout)	GAD-7 (Anxiety)	PHQ-9 (Depression)	EQ-5D LSS	EQ-VAS
Both vs. Control	0.29 [0.21, 0.37] d = 0.36 *p* < .001	2.17 [1.54, 2.79] d = 0.45 *p* < .001	2.33 [1.58, 3.07] d = 0.40 *p* < .001	0.25 [0.17, 0.33] d = 0.37 *p* < .001	-4.64 [-7.70, -1.58] d = -0.20 *p* < .001
Both vs. RZ Only	0.24 [0.15, 0.33] d = 0.30 *p* < .001	2.21 [1.46, 2.96] d = 0.43 *p* < .001	2.68 [1.79, 3.57] d = 0.45 *p* < .001	0.31 [0.21, 0.41] d = 0.45 *p* < .001	-7.48 [-11.13, -3.83] d = -0.33 *p* < .001
Both vs. COVID Only	0.11 [0.01, 0.21] d = 0.13 *p* = .019	0.93 [0.15, 1.72] d = 0.17 *p* = .012	1.06 [0.13, 2.00] d = 0.17 *p* = .018	0.16 [0.06, 0.26] d = 0.22 *p* < .001	-1.69 [-5.52, 2.15] d = -0.07 *p* = .670
COVID Only vs. Control	0.18 [0.10, 0.26] d = 0.26 *p* < .001	1.23 [0.55, 1.91] d = 0.28 *p* < .001	1.26 [0.45, 2.07] d = 0.23 *p* < .001	0.09 [0.00, 0.18] d = 0.15 *p* = .051	-2.95 [-6.27, 0.37] d = -0.13 *p* = .101
COVID Only vs. RZ Only	0.13 [0.04, 0.22] d = 0.19 *p* = .001	1.28 [0.48, 2.07] d = 0.28 *p* < .001	1.62 [0.68, 2.56] d = 0.31 *p* < .001	0.15 [0.05, 0.26] d = 0.29 *p* = .001	-5.79 [-9.66, -1.92] d = -0.25 *p* < .001
RZ Only vs. Control	0.05 [-0.02, 0.12] d = 0.07 *p* = .246	-0.05 [-0.68, 0.59] d = -0.01 *p* = .998	-0.36 [-1.11, 0.40] d = -0.07 *p* = .622	-0.06 [-0.15, 0.02] d = -0.11 *p* = .255	2.84 [-0.26, 5.94] d = 0.12 *p* = .086

Mean differences (MD) with 95% CI, Cohen’s d, and adjusted *p*-values are reported. Values reflect corrected post-hoc contrasts following one-way ANOVA

**Fig. 1:**
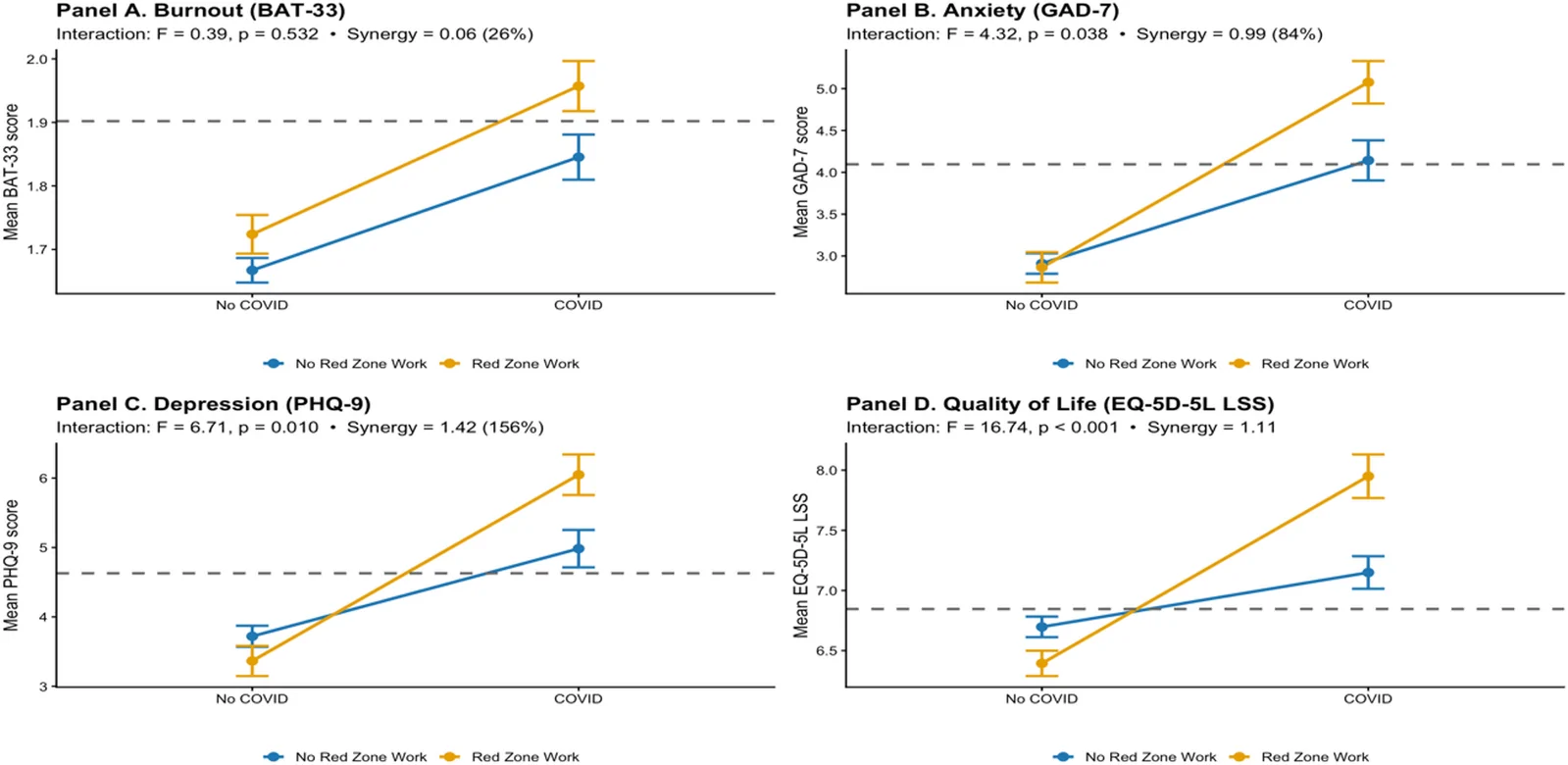
Comparison of observed and expected values for selected outcomes under an additive model of COVID-19 infection and Red Zone work. Expected values represent the sum of independent effects of each exposure under an additive assumption. Deviations of observed values from expected values indicate non-additive (interaction) effects. This figure complements, but does not replace, the formal interaction tests reported in Table 3. Synergy estimates are based on observed group means and are presented for illustrative purposes. All four outcomes are shown to allow comparison of additive and non-additive patterns across domains

Figure 1 illustrates the pattern of additive and non-additive effects across outcomes, complementing the formal interaction tests reported in Table 3. For burnout (BAT-33), mean scores increased with each exposure; however, the COVID × Red Zone interaction was not statistically significant. The observed dual-exposure mean (M = 1.96) was close to the additive prediction (M = 1.90), indicating an additive pattern. For anxiety and depression, positive deviations from additivity were observed. For GAD-7, the dual-exposure group (M = 5.08) exceeded the additive prediction (M = 4.10) by nearly one point (+ 0.98), consistent with a significant interaction. A similar pattern emerged for PHQ-9, where the observed mean (M = 6.05) was higher than the predicted additive value (M = 4.63), yielding a deviation of + 1.42-points from the additive prediction. For quality of life (EQ-5D-5 L LSS), the dual-exposure group also exceeded the additive prediction (M = 7.95 vs. 6.84), with a deviation of 1.11 points, corresponding to a significant interaction (F = 16.74, *p* < .001).

Table 5 presents logistic regression models predicting clinically significant anxiety (GAD-7 ≥ 10) and depression (PHQ-9 ≥ 10). Prior COVID-19 infection was the strongest independent predictor for both outcomes (Anxiety: OR = 2.33, 95% CI: 1.74–3.11, *p* < .001; Depression: OR = 1.89, 95% CI: 1.44–2.49, *p* < .001). Profession showed a strong independent association, with nurses demonstrating significantly lower odds of both anxiety (OR = 0.33, 95% CI: 0.20–0.54, *p* < .001) and depression (OR = 0.48, 95% CI: 0.30–0.77, *p* = .002) compared with physicians. Older age showed a modest protective association with both outcomes (OR = 0.97, *p* = .001 for anxiety; OR = 0.96, *p* < .001 for depression). Female sex, work experience, and higher education were not significantly associated with either outcome after full adjustment. Red Zone work was not significantly associated with either outcome (*p* = .142 for anxiety; *p* = .508 for depression). Region of residence was significantly associated with depression but not with anxiety. Model performance was acceptable (Nagelkerke R² = 0.149 for anxiety; R² = 0.127 for depression), consistent with expectations for population-based epidemiological models predicting clinical thresholds from exposure and sociodemographic variables. Overall, 253 participants (9.7%) met criteria for clinically significant anxiety (GAD-7 ≥ 10) and 291 (11.1%) for clinically significant depression (PHQ-9 ≥ 10).

**Table 5:** Predictors of Clinical Anxiety (GAD-7 ≥ 10) and Depression (PHQ-9 ≥ 10): Logistic Regression (*N* = 2,644)

Predictor	Anxiety OR [95% CI]	*p*	Depression OR [95% CI]	*p*
Female (1 = yes)	0.99 [0.60, 1.65]	0.984	0.75 [0.47, 1.18]	0.217
Age (years)	0.97 [0.94, 0.99]	0.001	0.96 [0.94, 0.98]	< 0.001
Work experience	1.01 [0.99, 1.03]	0.347	1.02 [1.00, 1.04]	0.131
Higher education	1.16 [0.79, 1.71]	0.438	1.31 [0.93, 1.85]	0.127
Profession	0.33 [0.20, 0.54]	< 0.001	0.48 [0.30, 0.77]	0.002
Had COVID-19 (1 = yes)	2.33 [1.74, 3.11]	< 0.001	1.89 [1.44, 2.49]	< 0.001
Worked in red zone (1 = yes)	1.24 [0.93, 1.64]	0.142	1.09 [0.84, 1.43]	0.508
Region	0.70 [0.48, 1.02]	0.064	0.69 [0.48, 0.99]	0.041
Model Fit	Nagelkerke R² = 0.149; AIC = 1568.8		Nagelkerke R² = 0.127; AIC = 1754.6	
Outcome prevalence	Anxiety 253 (9.7%)		Depression 291 (11.1%)	

**Fig. 2:**
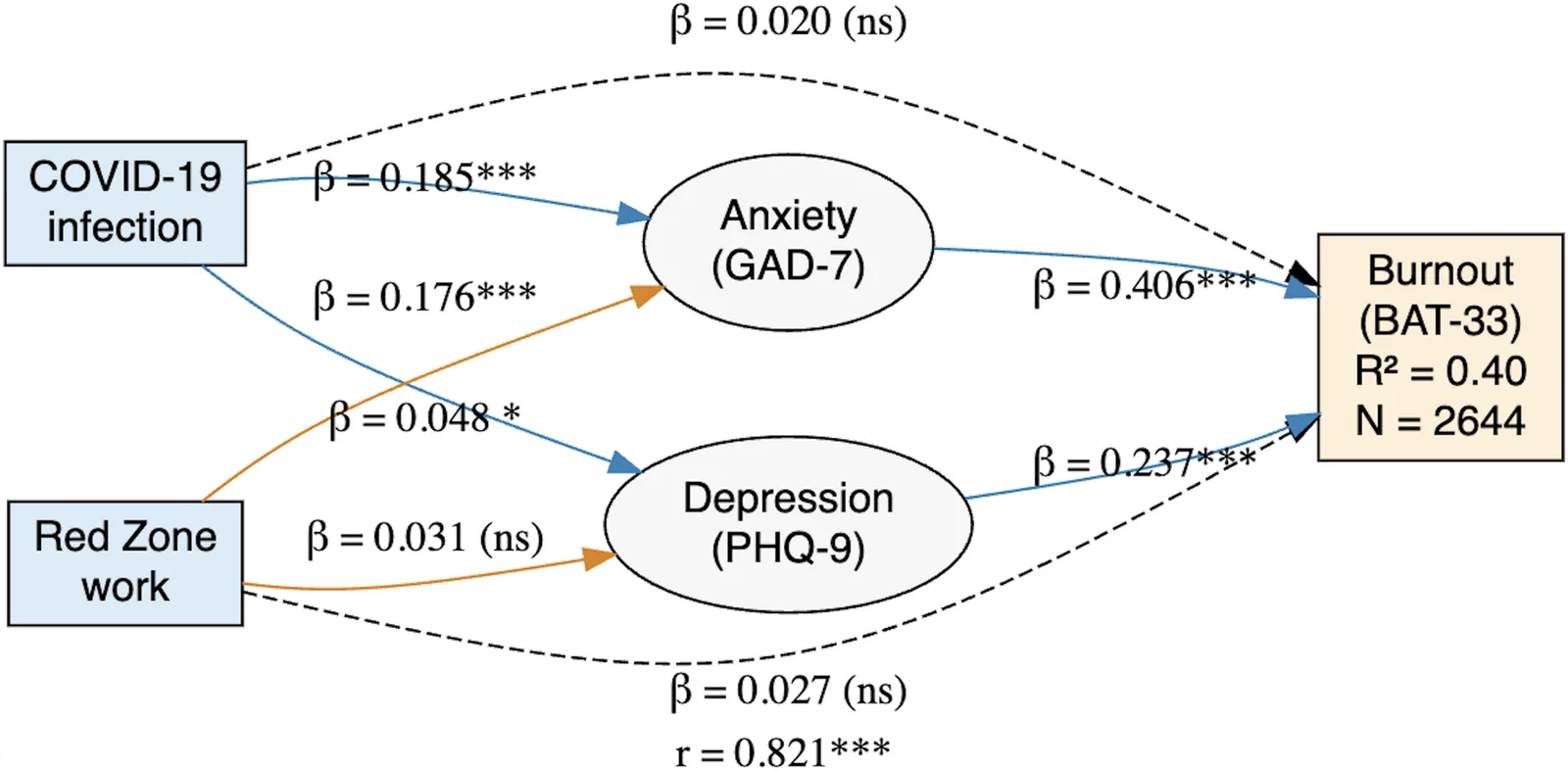
Parallel mediation model (path analysis) showing the pathways through which COVID-19 infection and Red Zone work influence burnout via anxiety (GAD-7) and depression (PHQ-9). Values on the paths are standardized coefficients. The model includes the covariance between the two mediators (GAD-7 ↔ PHQ-9). Significance levels: ***p < .001, **p < .01, *p < .05. Solid arrows indicate statistically significant paths (*p* < .05), whereas dashed arrows represent non-significant paths. Arrow direction represents the hypothesized relationships specified in the mediation model

Figure 2 presents the mediation model with standardised path coefficients. Table 6 summarises indirect and total effects with unstandardised estimates and bootstrap confidence intervals. Direct path coefficients are provided in Supplementary Table S2. After including both mediators, neither COVID-19 infection nor Red Zone work showed a significant direct association with burnout (COVID-19: std.β = 0.020, *p* = .233; Red Zone work: std.β = 0.027, *p* = .094). These results indicate that the associations between pandemic-related exposures and burnout are accounted for by anxiety and depression rather than by direct effects.

We identified significant indirect pathways through both mediators (Table 6). For COVID-19 infection, path a indicated higher anxiety (std. β = 0.185, *p* < .001) and depression (std.β = 0.176, *p* < .001). Path b showed that both anxiety (std.β = 0.406, *p* < .001) and depression (std.β = 0.237, *p* < .001) were strong predictors of burnout. These paths yielded a significant indirect effect of COVID-19 infection on burnout (B = 0.184, 95% CI 0.143–0.228; std.β = 0.117), with anxiety contributing more strongly than depression. For Red Zone work, the indirect effect was smaller but statistically significant (B = 0.041, 95% CI 0.007–0.078; std.β = 0.027), driven primarily by anxiety (std.β = 0.019), while the pathway through depression was not significant (std.β = 0.007, *p* = .133). These findings indicate that both exposures are indirectly associated with burnout through elevated psychological symptoms, although personal COVID-19 infection shows a substantially stronger indirect association than Red Zone work. The mediation model was specified as a fully observed path model without latent constructs. Because all structural paths and the covariance between mediators were freely estimated, the model was just-identified (df = 0). Consequently, global fit indices were perfect by definition (CFI = 1.00, TLI = 1.00, RMSEA = 0.00, SRMR = 0.00) and are not informative in this context. Model evaluation therefore focused on path coefficients and bootstrap confidence intervals.

**Table 6:** Indirect and total effects of COVID-19 infection and Red Zone work on burnout (BAT-33). Unstandardized estimates, standardized coefficients (β), and percentile bootstrap 95% confidence intervals (2,000 resamples) are reported

Predictor	Effect type	Estimate (unstd)	Std. β	95% CI (unstd)	*p*-value
COVID-19 infection	Indirect (total)	0.184	0.117	0.143–0.228	< 0.001
	Via Anxiety	0.118	0.075	0.087–0.155	< 0.001
	Via Depression	0.066	0.042	0.044–0.092	< 0.001
	Direct	0.031	0.020	–0.019–0.085	0.233
	Total effect	0.215	0.137	0.153–0.283	< 0.001
Red Zone work	Indirect (total)	0.041	0.027	0.007–0.078	0.023
	Via Anxiety	0.030	0.019	0.007–0.054	0.013
	Via Depression	0.011	0.007	–0.002–0.027	0.133
	Direct	0.041	0.027	–0.006–0.091	0.094
	Total effect	0.083	0.053	0.023–0.144	0.007

Taken together, these findings suggest that anxiety and depression are strongly associated with both pandemic-related exposures and burnout. Because of the cross-sectional design, these mediation findings should be interpreted as statistical decomposition of associations rather than evidence of temporal causality.

## Discussion

This study examined how personal COVID-19 infection and Red Zone work were associated with burnout, anxiety, and depression among healthcare workers in Kazakhstan 3–5 years after the onset of the pandemic. We found that both exposures were associated with elevated psychological symptoms, consistent with the health impairment process described in the JD–R model. The combined effects on burnout followed an additive pattern. In contrast, anxiety and depression demonstrated significant positive deviations from additivity. Workers exposed to both factors reported higher symptom levels than expected from independent effects. In mediation analyses, anxiety and depression accounted for 39.7% of the variance in burnout.

To situate our findings against international evidence, we compared symptom levels with published reports from a Dutch longitudinal cohort of healthcare workers (Lavell et al., 2025), which assessed mental health using the same instruments (PHQ-9, GAD-7) and reported subgroup-specific scores by SARS-CoV-2 exposure status. In the Dutch cohort, mean PHQ-9 scores ranged from 4.45 (95% CI: 4.07–4.84) in SARS-CoV-2-naïve workers to 5.54 (95% CI: 4.69–6.39) in those working in COVID-19 wards at the December 2021 pandemic peak, while mean GAD-7 scores ranged from 3.62 (95% CI: 3.28–3.97) in naïve workers to 4.52 (95% CI: 3.78–5.25) in ward workers. The overall mean scores in our sample (PHQ-9 = 4.36; GAD-7 = 3.55) closely matched the Dutch values for SARS-CoV-2-naïve workers and baseline pre-peak levels. However, the mean scores in our dual-exposed subgroup (PHQ-9 = 6.05; GAD-7 = 5.08) exceeded the peak pandemic values observed in Dutch COVID-19 ward workers, despite being measured 3–5 years after the acute phase. With respect to burnout, the mean BAT total score in our overall sample (M = 1.76 per item, BAT-33) was lower than values reported in representative healthy European working populations using the BAT-23 (pooled Netherlands, Belgium, and Finland: M = 2.10, SD = 0.67; Schaufeli et al., [43]) and in a multi-country sample from Australia, the Netherlands, South Africa, and the United States (M = 2.52, SD = 0.62; Redelinghuys & Morgan, [44]). The mean score in our dual-exposed subgroup (M = 1.96) was comparable to that observed in Italian healthcare workers during the pandemic using the BAT-12 (adjusted M = 1.98; [45]). Direct numerical comparison across these studies should be interpreted cautiously, as the BAT-23 and BAT-12 differ from the BAT-33 used here in item composition. Prevalence comparisons with other healthcare-worker cohorts further situate our findings within the international context.

Our findings sit between the lower symptom prevalence reported in European HCW cohorts and the higher levels documented in Sub-Saharan African settings (e.g., Otai et al., reported anxiety prevalence of 27.3% and depression prevalence of 57.6% among Ugandan healthcare workers during the pandemic) [46]. This intermediate position likely reflects the broader healthcare system context of Kazakhstan, while the differential pattern between our exposure subgroups remains the central empirical contribution of this study.

One aspect of our results is that burnout followed an additive pattern, while anxiety and depression showed positive deviations from additivity. We propose that differences in measurement windows may contribute to this divergence, although alternative explanations involving distinct underlying mechanisms cannot be excluded. Burnout, as captured by the BAT-33, is understood as a chronic syndrome that develops gradually through the cumulative accumulation of occupational stressors over months and years [31]. By its construction, this measure already integrates the temporal averaging of exposures, which may explain why the contributions of two stressors operating through partially distinct mechanisms (Red Zone work as an occupational demand, and personal COVID-19 infection as a personal health stressor) summed approximately additively. In contrast, the GAD-7 and PHQ-9 assess affective symptoms over the preceding two weeks [33, 34, 47] and are therefore more sensitive to the current state of an individual’s coping resources at the time of measurement. When coping reserves are already partially depleted by one stressor, the addition of a second stressor may produce a disproportionate increase in acute affective symptoms, in accordance with stress-sensitisation models proposing that prior stress exposure lowers the threshold for adverse affective responses to subsequent stressors [13, 14]. Under this interpretation, the divergent statistical patterns are not theoretically contradictory but reflect that burnout integrates stressors over a long temporal window, whereas current affective symptoms reflect more immediate resource availability. We emphasise that this account is a post-hoc theoretical interpretation rather than a confirmatory test, and that the cross-sectional design does not allow direct examination of temporal dynamics.

In adjusted models, Red Zone work showed limited independent associations with outcomes. We did not collect data on the duration, frequency, or intensity of Red Zone assignments, which precludes interpretation of the underlying mechanism.

The statistical decomposition indicated that anxiety and depression were strongly associated with both pandemic-related exposures and burnout, and that a substantial portion of the exposure–burnout association co-occurred with these affective symptoms. This is consistent with prior research showing strong associations between affective symptoms and burnout among healthcare workers [37, 48]. Because of the cross-sectional design, these constructs are best characterised as interrelated psychological states rather than as temporally ordered components. Several interpretations of this pattern remain plausible: anxiety and depression may co-occur with burnout, burnout may contribute to anxiety and depression rather than resulting from them, or these constructs may influence each other bidirectionally over time. In our mediation model, anxiety and depression statistically accounted for 39.7% of the variance in burnout (R²). Longitudinal research is needed to clarify the temporal direction of these associations.

The regional literature from Kazakhstan and Central Asia provides important context for interpreting the magnitude of psychological burden observed in our sample. Although healthcare worker mental health has received increasing research attention globally, few studies have focused on this region. Seisembekov et al. reported that 37.4% of healthcare workers experienced additional workload during the pandemic, while approximately one quarter indicated overtime work and insufficient staffing [20]. Additionally, Bolatov et al. (2025) documented substantial occupational burnout among healthcare workers across Central Asian healthcare systems, linking burnout to high workload, unfavorable work environments, and pandemic-related stressors such as social stigma and lack of appreciation [7]. Evidence from Kazakhstan shows heightened levels of anxiety and depression during the pandemic [49], and WHO reports describe significant emotional strain among the country’s healthcare workforce [50]. For example, research among emergency medical personnel in eastern Kazakhstan identified elevated levels of anxiety, depression, and insomnia among medical staff [51]. Additional research conducted among hospital nurses in Kazakhstan reported that nearly two-thirds of respondents (61.9%) exhibited signs of burnout, highlighting the substantial psychological burden experienced by nursing staff in clinical settings [52]. Similarly, studies conducted among frontline healthcare workers during the COVID-19 pandemic found that insufficient financial support was associated with higher levels of burnout, depression, and anxiety, highlighting the role of structural and organizational factors in shaping healthcare workers’ well-being [53]. Notably, our adjusted models showed that participants from southern regions had lower odds of depression but not of anxiety. These associations should be interpreted cautiously: the sample was heavily concentrated in the southern region (93.5%), and the observed regional effects may largely reflect underlying sample composition rather than true geographic differences in mental health. As shown in the international comparisons above, our overall symptom prevalence is broadly consistent with post-pandemic estimates among healthcare workers in other settings. Within our sample, however, the dual-exposed subgroup showed approximately 2-fold higher prevalence of clinically significant anxiety and depression compared with the Control group. This differential pattern between exposure subgroups represents the principal empirical contribution of our study, identifying a subgroup of healthcare workers who report substantially elevated psychological symptoms relative to colleagues without these exposures.

That psychological symptoms appear elevated in specific exposure subgroups 3–5 years after the pandemic onset suggests that short-term, acute-phase interventions alone may be insufficient for these workers. Our findings indicate that workers reporting both personal COVID-19 history and Red Zone deployment represent a subgroup that warrants continued monitoring and further longitudinal investigation, rather than serving as a basis for immediate reallocation of limited mental health resources. Because anxiety, depression, and burnout were strongly interrelated in our sample, interventions addressing affective symptoms may also be relevant to burnout. Future longitudinal studies using objective exposure verification are needed before specific prioritization recommendations can be substantiated.

This study has several limitations. First, the cross-sectional design does not allow determination of temporal relationships between exposures and outcomes. In particular, it cannot be established whether anxiety and depressive symptoms preceded burnout or emerged as consequences of occupational strain. Second, although analyses adjusted for several demographic and occupational variables, residual confounding cannot be fully excluded. Factors such as workplace support, institutional policies, or pre-existing mental health conditions were not directly measured and may have influenced the observed associations. Hospital type and shift characteristics were not collected and could not be included as covariates, which represents an additional source of residual confounding. Third, the use of convenience sampling through open professional networks introduces potential selection bias. Because participation was voluntary and the response rate could not be calculated, healthcare workers who self-selected into the study may differ systematically from non-respondents on characteristics related to mental health, which may have influenced the observed associations. Fourth, the sample consisted predominantly of nurses (91.7%), which reflects workforce composition in many healthcare settings but limits the generalizability of findings to physicians and other healthcare professions. The differential patterns observed between nurses and physicians in supplementary analyses (Table S1) suggest that occupational role may moderate the relationship between pandemic exposures and psychological outcomes. Future research should ensure adequate representation of diverse healthcare professions to enable more robust comparative analyses. Fifth, the retrospective assessment of COVID-19 infection and Red Zone work (exposures occurring 3–5 years before data collection) introduces a substantial risk of differential recall bias. Participants currently experiencing higher levels of burnout, anxiety, or depression may engage in “effort after meaning,” whereby current psychological distress influences the vividness and accessibility of memories of past stressors, leading to systematically amplified recall of pandemic-era exposures compared to participants with lower symptom levels. Because this bias is linked to the outcomes themselves, it operates non-randomly and systematically inflates the observed associations between pandemic exposures and current psychological outcomes. Consequently, the odds ratios, path coefficients, and interaction estimates reported in this study should be interpreted as likely upper-bound estimates rather than unbiased effect sizes. Our study did not employ objective verification of exposures through administrative infection records, official duty rosters, or hospital staffing logs, nor did it use structured recall-enhancing techniques. We therefore cannot rule out, and explicitly acknowledge, that the reported effect estimates are likely upwardly biased. Future research should incorporate objective exposure verification (e.g., serological records, employment registries) to provide unbiased estimates of these associations. Sixth, Red Zone work was measured as a binary exposure variable (yes/no), without information on duration, frequency, or intensity of deployment. This precludes examination of dose-response relationships and may have contributed to attenuation of observed effects through within-group heterogeneity. Seventh, while our path analysis provides statistical evidence consistent with anxiety and depression as interrelated symptoms, the cross-sectional design precludes definitive conclusions about temporal ordering or causality. Longitudinal research is needed to establish whether anxiety and depression clarify the temporal sequence or whether these relationships are bidirectional.

## Conclusions

This cross-sectional study observed that self-reported COVID-19 infection and Red Zone work remained statistically associated with the mental health of healthcare workers 3–5 years after the pandemic. While burnout followed an additive pattern, anxiety and depression showed positive deviations from additivity, with the dual-exposed subgroup showing approximately 2-fold higher prevalence of clinically significant symptoms compared with unexposed colleagues. Anxiety and depression were strongly associated with burnout in this sample, although the cross-sectional and retrospective design does not permit conclusions about temporal sequence or causal direction. Healthcare workers reporting both COVID-19 infection and Red Zone experience may represent a subgroup that warrants continued monitoring in future longitudinal research with objective exposure verification, before specific clinical or policy recommendations can be advanced.

## Supplementary Information

Supplementary Material 1.

## Data Availability

Data are available from the corresponding author upon reasonable request.

## References

[1] World Health Organization. Burnout in Hospital-Based Healthcare Workers during COVID-19. Geneva. 2021. 10.47326/ocsat.2021.02.46.1.0.

[2] Davis HE, McCorkell L, Vogel JM, Topol EJ, Long COVID. major findings, mechanisms and recommendations. Nat Rev Microbiol. 2023;21:133–46. 10.1038/s41579-022-00846-2. PMC9839201 36639608

[3] Hobfoll SE, Halbesleben J, Neveu J-P, Westman M Conservation of resources in the organizational context: the reality of resources and their consequences. Annu Rev Organ Psychol Organ Behav. 2018;5:103–28. 10.1146/annurev-orgpsych-032117-104640.

[4] Hobfoll SE. The influence of culture, community, and the nested-self in the stress process: Advancing Conservation of Resources theory. Appl Psychol Int Rev. 2001;50:337–70. 10.1111/1464-0597.00062.

[5] Kisely S, Warren N, McMahon L, Dalais C, Henry I, Siskind D. Occurrence, prevention, and management of the psychological effects of emerging virus outbreaks on healthcare workers: rapid review and meta-analysis. BMJ. 2020;369:m1642. 10.1136/bmj.m1642. PMC7199468 32371466

[6] Sun H, Zhang T, Wang X, Wang C, Zhang M, Song H. The occupational burnout among medical staff with high workloads after the COVID-19 and its association with anxiety and depression. Front Public Health. 2023;11. 10.3389/fpubh.2023.1270634. PMC10639132 37954047

[7] Bolatov AK, Brimkulov N, Jarylkasynova G, Taalaikanova A, Yuldashova R, Kodirova S, et al. Occupational burnout among healthcare workers in Central Asia during the COVID-19 pandemic. Sci Rep. 2025;15:12432. 10.1038/s41598-025-96905-8. PMC11992192 40216936

[8] Sipos D, Goyal R, Zapata T. Addressing burnout in the healthcare workforce: current realities and mitigation strategies. Lancet Reg Health – Eur. 2024;42. 10.1016/j.lanepe.2024.100961. PMC11281910 39070752

[9] Bakker AB, Demerouti E. The Job Demands-Resources model: state of the art. J Manag Psychol. 2007;22:309–28. 10.1108/02683940710733115.

[10] Demerouti E, Sanz Vergel A. Burnout and Work Engagement: The JD-R Approach. Annu Rev Organ Psychol Organ Behav. 2014;1. 10.1146/annurev-orgpsych-031413-091235.

[11] Rothman KJ, Causes. Am J Epidemiol. 1976;104:587–92. 10.1093/oxfordjournals.aje.a112335. 998606

[12] Zhu H, Yang X, Xie S, Zhou J. Prevalence of burnout and mental health problems among medical staff during the COVID-19 pandemic: a systematic review and meta-analysis. BMJ Open. 2023;13:e061945. 10.1136/bmjopen-2022-061945. PMC10360428 37474193

[13] Hammen C. Stress and Depression. Annu Rev Clin Psychol. 2005;1(1, 2005):293–319. 10.1146/annurev.clinpsy.1.102803.143938. 17716090

[14] McLaughlin KA, Conron KJ, Koenen KC, Gilman SE. Childhood adversity, adult stressful life events, and risk of past-year psychiatric disorder: a test of the stress sensitization hypothesis in a population-based sample of adults. Psychol Med. 2010;40:1647–58. 10.1017/S0033291709992121. PMC2891275 20018126

[15] Nikunlaakso R, Reuna K, Selander K, Oksanen T, Laitinen J. Synergistic Interaction between Job Stressors and Psychological Distress during the COVID-19 Pandemic: A Cross-Sectional Study. Int J Environ Res Public Health. 2022;19:13991. 10.3390/ijerph192113991. PMC9656466 36360868

[16] Prasad K, McLoughlin C, Stillman M, Poplau S, Goelz E, Taylor S, et al. Prevalence and correlates of stress and burnout among U.S. healthcare workers during the COVID-19 pandemic: A national cross-sectional survey study. eClinicalMedicine. 2021;35. 10.1016/j.eclinm.2021.100879. PMC8141518 34041456

[17] Sexton JB, Adair KC, Proulx J, Profit J, Cui X, Bae J, et al. Emotional Exhaustion Among US Health Care Workers Before and During the COVID-19 Pandemic, 2019–2021. JAMA Netw Open. 2022;5:e2232748. 10.1001/jamanetworkopen.2022.32748. PMC9494188 36129705

[18] Hendrickson RC, McCall CA, Rosser AF, Pagulayan KF, Chang BP, Sano ED, et al. The relative contribution of COVID-19 infection versus COVID-19 related occupational stressors to insomnia in healthcare workers. Sleep Med X. 2023;5:100067. 10.1016/j.sleepx.2023.100067. PMC9979693 36923965

[19] Partners In Health: Kazakhstan. Partners In Health. 2025. https://www.pih.org/country/kazakhstan. Accessed 24 Nov 2025.

[20] Seisembekov T, Bolatov A, Brimkulov N, Jarylkasynova G, Taalaikanova A, Yuldashova R et al. Mental health and well-being of healthcare workers in Central Asia. 2024. 10.12688/f1000research.153832.1 PMC11992192 40216936

[21] Demerouti E, Bakker AB, Nachreiner F, Schaufeli WB. The job demands-resources model of burnout. J Appl Psychol. 2001;86:499–512. 11419809

[22] Leiter MP, Maslach C. Nurse turnover: the mediating role of burnout. J Nurs Manag. 2009;17:331–9. 10.1111/j.1365-2834.2009.01004.x. 19426369

[23] NRCHD. Ministry of Health of the Republic of Kazakhstan. Health of the Population of the Republic of Kazakhstan and the Activities of Healthcare Organizations in 2024. Statistical Yearbook. Astana; 2025.

[24] Faul F, Erdfelder E, Lang A-G, Buchner A. G*Power 3: A flexible statistical power analysis program for the social, behavioral, and biomedical sciences. Behav Res Methods. 2007;39:175–91. 10.3758/BF03193146. 17695343

[25] Glushkova N, Ivankov A, Trenina V, Oshibayeva A, Kalmatayeva Z, Temirbekova Z, et al. Post-conflict acute stress reactions in Kazakhstan in the aftermath of January 2022 unrests: A national survey. Heliyon. 2023;9. 10.1016/j.heliyon.2023.e21065. PMC10641126 37964844

[26] Ibrayeva Z, Aldyngurov D, Myssayev A, Meirmanov S, Zhanaspayev M, Khismetova Z, et al. Depression, Anxiety and Somatic Distress in Domestic and International Undergraduate Medical Students in Kazakhstan. Iran J Public Health. 2018;47:919–21. PMC6077647 30087881

[27] Semenova Y, Pivina L, Manatova A, Bjørklund G, Glushkova N, Belikhina T, et al. Mental distress in the rural Kazakhstani population exposed and non-exposed to radiation from the Semipalatinsk Nuclear Test Site. J Environ Radioact. 2019;203:39–47. 10.1016/j.jenvrad.2019.02.013. 30849560

[28] Mergenova G, Davis A, Gilbert L, El-Bassel N, Terlikbayeva A, Primbetova S, et al. Mental health and cognition in relation to adherence to antiretroviral therapy among people living with HIV in Kazakhstan: a cross-sectional study. J Int AIDS Soc. 2024;27:e26320. 10.1002/jia2.26320. PMC11258456 39030875

[29] Turgunova L, Laryushina Y, Turmukhambetova A, Koichubekov B, Sorokina M, Korshukov I. The Incidence of Depression among the Population of Central Kazakhstan and Its Relationship with Sociodemographic Characteristics. Behav Neurol. 2017;2017:2584187. 10.1155/2017/2584187. PMC5676366 29209102

[30] Terloyeva D, Nugmanova Z, Akhmetova G, Akanov A, Patel N, Lazariu V, et al. Untreated depression among persons living with human immunodeficiency virus in Kazakhstan: A cross-sectional study. PLoS ONE. 2018;13:e0193976. 10.1371/journal.pone.0193976. PMC5873996 29590151

[31] Schaufeli WB, Desart S, De Witte H. Burnout Assessment Tool (BAT)—Development, Validity, and Reliability. Int J Environ Res Public Health. 2020;17:9495. 10.3390/ijerph17249495. PMC7766078 33352940

[32] Schaufeli W, Witte HD, Desart S. Manual Burnout Assessment Tool (BAT)–Version 2.0. 2020.

[33] Spitzer RL, Kroenke K, Williams JBW, Löwe B. A Brief Measure for Assessing Generalized Anxiety Disorder: The GAD-7. Arch Intern Med. 2006;166:1092–7. 10.1001/archinte.166.10.1092. 16717171

[34] Kroenke K, Spitzer RL, Williams JB. The PHQ-9: validity of a brief depression severity measure. J Gen Intern Med. 2001;16:606–13. 10.1046/j.1525-1497.2001.016009606.x. PMC1495268 11556941

[35] Herdman M, Gudex C, Lloyd A, Janssen MF, Kind P, Parkin D, et al. Development and preliminary testing of the new five-level version of EQ-5D (EQ-5D-5L). Qual Life Res. 2011;20:1727–36. 10.1007/s11136-011-9903-x. PMC3220807 21479777

[36] Feng Y-S, Jiang R, Pickard AS, Kohlmann T, Combining. EQ-5D-5L items into a level summary score: demonstrating feasibility using non-parametric item response theory using an international dataset. Qual Life Res. 2022;31:11–23. 10.1007/s11136-021-02922-1. PMC8800896 34236579

[37] Feng Q, Zhou T, Chen C, Xia X. The complex relationship between the digital divide, social capital, and mental health among older adults: a multi-method path decomposition. Front Psychol. 2025;16. 10.3389/fpsyg.2025.1670203. PMC12640981 41293096

[38] Liu Y, Wan C, Xi X. Measurement properties of the EQ-5D-5L in sub-health: evidence based on primary health care workers in China. Health Qual Life Outcomes. 2023;21:22. 10.1186/s12955-023-02105-1. PMC9996950 36890491

[39] Hołownia-Voloskova M, Tarbastaev A, Golicki D. Population norms of health-related quality of life in Moscow, Russia: the EQ-5D-5L-based survey. Qual Life Res. 2021;30:831–40. 10.1007/s11136-020-02705-0. PMC7952340 33237551

[40] Janssen MF, Pickard AS, Golicki D, Gudex C, Niewada M, Scalone L, et al. Measurement properties of the EQ-5D-5L compared to the EQ-5D-3L across eight patient groups: a multi-country study. Qual Life Res Int J Qual Life Asp Treat Care Rehabil. 2013;22:1717–27. 10.1007/s11136-012-0322-4. PMC3764313 23184421

[41] Cohen J. Statistical power analysis for the behavioral sciences. 2nd. ed., reprint. New York: Psychology Press; 2009.

[42] Maxwell SE, Cole DA. Bias in cross-sectional analyses of longitudinal mediation. Psychol Methods. 2007;12:23–44. 10.1037/1082-989X.12.1.23. 17402810

[43] Schaufeli WB, De Witte H, Hakanen JJ, Kaltiainen J, Kok R How to assess severe burnout? Cutoff points for the Burnout Assessment Tool (BAT) based on three European samples. Scand J Work Environ Health. 2023;49:293–302. 10.5271/sjweh.4093. PMC10713992 37042446

[44] Redelinghuys K, Morgan B. Psychometric properties of the Burnout Assessment Tool across four countries. BMC Public Health. 2023;23:824. 10.1186/s12889-023-15604-z. PMC10161461 37143022

[45] Mazzetti G, Consiglio C, Santarpia FP, Borgogni L, Guglielmi D, Schaufeli WB. Italian Validation of the 12-Item Version of the Burnout Assessment Tool (BAT-12). Int J Environ Res Public Health. 2022;19:8562. 10.3390/ijerph19148562. PMC9322735 35886414

[46] Otai J, Ekakoro N, Masaba H, Nvumetta R. Prevalence and predictors of stress, anxiety, and depression among healthcare workers during the COVID-19 pandemic at a mental referral hospital in Kampala, Uganda. BMC Public Health. 2025;25:3386. 10.1186/s12889-025-24233-7. PMC12506417 41063028

[47] Löwe B, Decker O, Müller S, Brähler E, Schellberg D, Herzog W, et al. Validation and standardization of the Generalized Anxiety Disorder Screener (GAD-7) in the general population. Med Care. 2008;46:266–74. 10.1097/MLR.0b013e318160d093. 18388841

[48] Chen D, Ni Y, Lu J, Wang Y, Qi Q, Zhai H. Examining the impact of perceived stress, anxiety, and resilience on depression among medical staff after COVID-19 quarantine: a chain mediation analysis. Front Public Health. 2023;11. 10.3389/fpubh.2023.1250623. PMC10549932 37799150

[49] Mergenova G, Rosenthal SL, Myrkassymova A, Bukharbayeva A, Iskakova B, Izekenova A, et al. The COVID-19 pandemic and mental health in Kazakhstan. Camb Prisms Glob Ment Health. 2023;10:e52. 10.1017/gmh.2023.46. PMC10579662 37854418

[50] World Health Organization. Protecting the mental health of health workers: hope and resilience in Kazakhstan. https://www.who.int/europe/news/item/11-10-2021-protecting-the-mental-health-of-health-workers-hope-and-resilience-in-kazakhstan. Accessed 4 Dec 2025.

[51] Kussainova DK, Orazalina AS, Khismetova ZA, Serikova-Esengeldina D, Khamidullina ZG, Akhmetova KM, et al. Prevalence of Anxiety, Depression, and Insomnia Among Medical Workers in Emergency Medical Services in Eastern Kazakhstan. Int J Environ Res Public Health. 2025;22:407. 10.3390/ijerph22030407. PMC11942404 40238520

[52] Maxudova M, Ospanova D, Stavropoulou A, Alibekova L, Sultanova G, Veklenko G, et al. Burnout Among Hospital Nurses in Kazakhstan. Nurs Rep. 2025;15:92. 10.3390/nursrep15030092. PMC11946353 40137665

[53] Bolatov A, Baikanova R, Igenbayeva B, Seisembekov T. Health care providers have faced the pandemic altruistically, but financial support is important for their well-being and motivation to work. Public Health. 2025;240:119–24. 10.1016/j.puhe.2025.01.023. 39904063

