# Supplementary material for: Long-term mental health outcomes among healthcare workers following COVID-19 infection and frontline work: evidence of additive and non-additive patterns in Kazakhstan
Source: BMC Public Health. 2026 Jun 25;26:3177. doi: 10.1186/s12889-026-28195-2 (PMC13729439; doi:10.1186/s12889-026-28195-2)
Supplement: Supplementary file 1 [file 12889_2026_28195_MOESM1_ESM.docx]

**Supplementary materials**

**Table S1** Stratified linear regression analyses among doctors and nurses.

Model 1 includes age, sex, years in healthcare, history of COVID-19 infection, and work in a COVID-19 Red Zone. Model 2 additionally includes anxiety (GAD-7 total score) and depression (PHQ-9 total score). Estimates are unstandardized regression coefficients with standard errors (SE), 95% confidence intervals (CI), and two-sided p-values. ΔR² and F-tests indicate improvement in model fit after inclusion of anxiety and depression. Female sex is coded as 1 (reference: male).

| Group | Term | Model 1: Estimate (SE) | Model 1: p | Model 1: 95% CI | Model 2: Estimate (SE) | Model 2: p | Model 2: 95% CI |
| --- | --- | --- | --- | --- | --- | --- | --- |
| Doctors | Intercept | 2.180 (0.520) | <.001 | [1.155; 3.204] | 1.322 (0.336) | <.001 | [0.660; 1.985] |
| Doctors | Age (years) | -0.021 (0.018) | 0.253 | [-0.056; 0.015] | -0.001 (0.012) | 0.949 | [-0.023; 0.022] |
| Doctors | Female (1=yes) | -0.289 (0.154) | 0.063 | [-0.592; 0.015] | -0.178 (0.099) | 0.074 | [-0.372; 0.017] |
| Doctors | Years in healthcare | 0.028 (0.017) | 0.098 | [-0.005; 0.061] | 0.002 (0.011) | 0.842 | [-0.019; 0.024] |
| Doctors | Had COVID-19 (1=yes) | 0.319 (0.156) | 0.042 | [0.012; 0.627] | -0.006 (0.102) | 0.955 | [-0.207; 0.196] |
| Doctors | Worked in Red zone (1=yes) | 0.934 (0.145) | <.001 | [0.648; 1.220] | 0.254 (0.101) | 0.013 | [0.055; 0.453] |
| Doctors | Anxiety (GAD-7 total) | |  |  | 0.052 (0.017) | 0.002 | [0.019; 0.084] |
| Doctors | Depression (PHQ-9 total) | |  |  | 0.069 (0.014) | <.001 | [0.041; 0.096] |
| Nurses | Intercept | 1.849 (0.086) | <.001 | [1.681; 2.018] | 1.451 (0.073) | <.001 | [1.308; 1.593] |
| Nurses | Age (years) | -0.005 (0.002) | 0.003 | [-0.009; -0.002] | -0.001 (0.002) | 0.585 | [-0.004; 0.002] |
| Nurses | Female (1=yes) | -0.018 (0.074) | 0.804 | [-0.163; 0.126] | -0.034 (0.061) | 0.576 | [-0.155; 0.086] |
| Nurses | Years in healthcare | 0.004 (0.002) | 0.047 | [0.000; 0.007] | 0.002 (0.002) | 0.199 | [-0.001; 0.005] |
| Nurses | Had COVID-19 (1=yes) | 0.157 (0.031) | <.001 | [0.095; 0.218] | 0.031 (0.026) | 0.246 | [-0.021; 0.082] |
| Nurses | Worked in Red zone (1=yes) | 0.008 (0.030) | 0.798 | [-0.052; 0.067] | 0.007 (0.025) | 0.797 | [-0.043; 0.056] |
| Nurses | Anxiety (GAD-7 total) | |  |  | 0.061 (0.004) | <.001 | [0.052; 0.069] |
| Nurses | Depression (PHQ-9 total) | |  |  | 0.026 (0.004) | <.001 | [0.019; 0.033] |
| Doctors | Model comparison (ΔR² = 0.446, F = 155.75, p = <.001) |  |  |  |  |  |  |
| Nurses | Model comparison (ΔR² = 0.303, F = 536.06, p = <.001) |  |  |  |  |  |  |
|  |  |  |  |  |  |  |  |

**Table S2** Direct effects from the parallel mediation model predicting burnout (BAT-33). Unstandardized regression coefficients and standardized estimates (β) are reported

| Predictor → Outcome | Estimate (unstd) | Std. β | p-value | 95% CI (unstd) |
| --- | --- | --- | --- | --- |
| COVID → Anxiety (GAD-7) | 1.850 | 0.185 | < .001 | 1.478 – 2.265 |
| COVID → Depression (PHQ-9) | 2.093 | 0.176 | < .001 | 1.625 – 2.557 |
| COVID → Burnout (BAT-33) | 0.031 | 0.020 | 0.233 | –0.019 – 0.085 |
| Red Zone → Anxiety (GAD-7) | 0.471 | 0.048 | 0.011 | 0.110 – 0.836 |
| Red Zone → Depression (PHQ-9) | 0.360 | 0.031 | 0.105 | –0.076 – 0.789 |
| Red Zone → Burnout (BAT-33) | 0.041 | 0.027 | 0.094 | –0.006 – 0.091 |
| Anxiety → Burnout | 0.064 | 0.406 | < .001 | 0.052 – 0.076 |
| Depression → Burnout | 0.031 | 0.237 | < .001 | 0.021 – 0.041 |
| Anxiety ↔ Depression (covariance) | 21.253 | 0.821 | < .001 | 19.203 – 23.178 |

**Table S3** Characteristics of participants with uncertain COVID-19 infection status (excluded from factorial analysis).

Group comparisons between included (n = 2,715) and excluded participants (n = 447) are presented. Values are mean ± SD or n (%). Continuous variables were compared using Wilcoxon rank-sum tests, and categorical variables using Pearson’s χ² tests. Although small differences in psychological symptom scores were observed, demographic characteristics were comparable between groups, suggesting limited selection bias.

| Variable | Included in factorial analysis N = 2,715*^1^* | Uncertain COVID (excluded) N = 447*^1^* | p-value*^2^* |
| --- | --- | --- | --- |
| Age | 40 ± 13 | 41 ± 12 | 0.2 |
| Female | 2,548 (94%) | 410 (92%) | 0.084 |
| Married | 1,552 (57%) | 257 (57%) | 0.9 |
| BAT_total | 1.76 ± 0.75 | 1.82 ± 0.72 | 0.020 |
| GAD7_total | 3.5 ± 4.8 | 3.9 ± 4.7 | 0.002 |
| PHQ9_total | 4.3 ± 5.7 | 5.0 ± 5.6 | <0.001 |

**Table S4** Internal consistency of EQ-5D-5L dimensions (N=2,715)

| Metric | Value |
| --- | --- |
| Cronbach’s α | 0.84 |
| 95% CI | 0.83–0.85 |
| Average inter-item correlation | 0.51 |
| Item-total correlations | 0.65–0.76 |
